# *N*-(2,6-Dichloro­phen­yl)-2-(naphthalen-1-yl)acetamide

**DOI:** 10.1107/S1600536812014869

**Published:** 2012-04-13

**Authors:** Hoong-Kun Fun, Ching Kheng Quah, Prakash S. Nayak, B. Narayana, B. K. Sarojini

**Affiliations:** a X-ray Crystallography Unit, School of Physics, Universiti Sains Malaysia, 11800 USM, Penang, Malaysia; b Department of Studies in Chemistry, Mangalore University, Mangalagangotri 574 199, India; c Department of Chemistry, P. A. College of Engineering, Nadupadavu, Mangalore 574 153, India

## Abstract

In the title compound, C_18_H_13_Cl_2_NO, the naphthalene ring system and the benzene ring form dihedral angles of 74.73 (13) and 62.53 (16)°, respectively, with the acetamide grouping [maximum deviation = 0.005 (3) Å]. The naphthalene ring system forms a dihedral angle of 75.14 (13)° with the benzene ring. In the crystal, mol­ecules are linked by N—H⋯O hydrogen bonds, forming *C*(4) chains propagating in [010]. The O atom also accepts two C—H⋯O inter­actions.

## Related literature

For related structures, see: Fun *et al.* (2010 ▶, 2011*a* ▶,*b* ▶). For the stability of the temperature controller used in the data collection, see: Cosier & Glazer (1986 ▶).
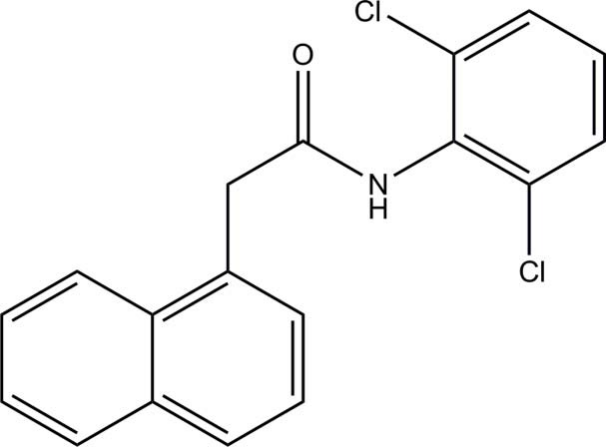


## Experimental

### 

#### Crystal data

C_18_H_13_Cl_2_NO
*M* *_r_* = 330.19
Monoclinic, 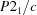

*a* = 13.1918 (13) Å
*b* = 4.7199 (5) Å
*c* = 24.878 (2) Å
β = 103.127 (3)°
*V* = 1508.5 (3) Å^3^
*Z* = 4
Mo *K*α radiation
μ = 0.43 mm^−1^
*T* = 100 K
0.38 × 0.13 × 0.08 mm

#### Data collection

Bruker SMART APEXII CCD diffractometer
Absorption correction: multi-scan (*SADABS*; Bruker, 2009 ▶) *T*_min_ = 0.853, *T*_max_ = 0.968
13545 measured reflections
4397 independent reflections
3245 reflections with *I* > 2σ(*I*)
*R*_int_ = 0.055

#### Refinement

*R*[*F* ^2^ > 2σ(*F* ^2^)] = 0.068
*wR*(*F* ^2^) = 0.168
*S* = 1.08
4397 reflections
203 parameters
H atoms treated by a mixture of independent and constrained refinement
Δρ_max_ = 0.53 e Å^−3^
Δρ_min_ = −0.56 e Å^−3^

### 

Data collection: *APEX2* (Bruker, 2009 ▶); cell refinement: *SAINT* (Bruker, 2009 ▶); data reduction: *SAINT*; program(s) used to solve structure: *SHELXTL* (Sheldrick, 2008 ▶); program(s) used to refine structure: *SHELXTL*; molecular graphics: *SHELXTL*; software used to prepare material for publication: *SHELXTL* and *PLATON* (Spek, 2009 ▶).

## Supplementary Material

Crystal structure: contains datablock(s) global, I. DOI: 10.1107/S1600536812014869/hb6731sup1.cif

Structure factors: contains datablock(s) I. DOI: 10.1107/S1600536812014869/hb6731Isup2.hkl

Supplementary material file. DOI: 10.1107/S1600536812014869/hb6731Isup3.cml

Additional supplementary materials:  crystallographic information; 3D view; checkCIF report

## Figures and Tables

**Table 1:** Hydrogen-bond geometry (Å, °)

*D*—H⋯*A*	*D*—H	H⋯*A*	*D*⋯*A*	*D*—H⋯*A*
N1—H1*N*1⋯O1^i^	0.84 (4)	2.00 (4)	2.823 (3)	165 (3)
C8—H8*A*⋯O1^i^	0.99	2.37	3.242 (4)	146
C8—H8*B*⋯O1^ii^	0.99	2.53	3.488 (4)	163

Symmetry codes: (i) 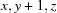; (ii) 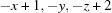.

**Fig. 1.**
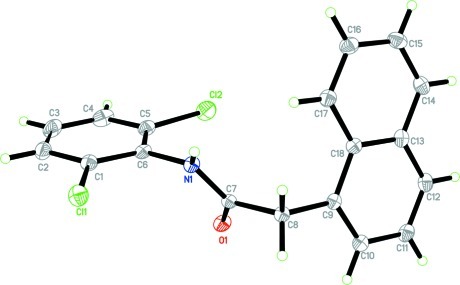
The molecular structure of the title compound showing 50% probability displacement ellipsoids for non-H atoms.

**Fig. 2.**
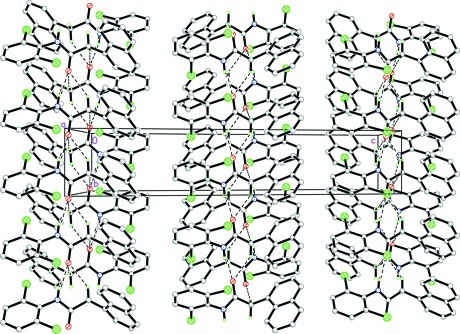
The crystal structure of the title compound, viewed along the a axis. H atoms not involved in hydrogen bonds (dashed lines) have been omitted for clarity.

## supplementary crystallographic information

### Comment

In continuation of our work on synthesis and structures of amides (Fun *et al.*, 2010, 2011*a*,*b*) we report herein the crystal structure of the title compound.

The molecular structure is shown in Fig. 1. Bond lengths are comparable to related structures (Fun *et al.*, 2010, 2011*a*,*b*). The naphthalene ring system (C9-C18, maximum deviation of 0.017 (3) Å at atom C9) and the benzene ring (C1-C6) form dihedral angles of 74.73 (13) and 62.53 (16)°, respectively, with the acetamide moiety (O1/N1/C7/C8, maximum deviation of 0.005 (3) Å at atom C7). The naphthalene ring system forms a dihedral angle of 75.14 (13)° with the benzene ring.

In the crystal, Fig. 2, molecules are linked *via* N1–H1N1···O1, C8–H8A···O1 and C8–H8B···O1 hydrogen bonds (Table 1) into two-molecule-thick chains along [010].

### Experimental

1-Naphthalene acetic acid (0.186 g, 1 mmol) and 2,6-dichloroaniline (0.162 g, 1 mmol), 1-ethyl-3-(3-dimethylaminopropyl)-carbodiimide hydrochloride (1.0 g, 0.01 mol) and were dissolved in dichloromethane (20ml). The mixture was stirred in presence of triethylamine at 273 K for about 3 h. The contents were poured into 100 ml of ice-cold aqueous hydrochloric acid with stirring, which was extracted thrice with dichloromethane. Organic layer was washed with saturated NaHCO_3_ solution and brine solution, dried and concentrated under reduced pressure to give the title compound. Colourless needles were grown from *N,N*-dimethyl formamide solution by the slow evaporation method (*m.p.*: 463K).

### Refinement

Atom H1N1 was located in a difference Fourier map and refined freely with N1-H1N1 = 0.85 (4) Å. The remaining H atoms were positioned geometrically and refined using a riding model with C–H = 0.95 or 0.99 Å and *U*_iso_(H) = 1.2 *U*_eq_(C).

### Crystal data

**Table:**

C_18_H_13_Cl_2_NO	*F*(000) = 680
*M**_r_* = 330.19	*D*_x_ = 1.454 Mg m^−^^3^
Monoclinic, *P*2_1_/*c*	Mo *K*α radiation, λ = 0.71073 Å
Hall symbol: -P 2ybc	Cell parameters from 3979 reflections
*a* = 13.1918 (13) Å	θ = 3.2–30.0°
*b* = 4.7199 (5) Å	µ = 0.43 mm^−^^1^
*c* = 24.878 (2) Å	*T* = 100 K
β = 103.127 (3)°	Needle, colourless
*V* = 1508.5 (3) Å^3^	0.38 × 0.13 × 0.08 mm
*Z* = 4	

### Data collection

**Table:**

Bruker SMART APEXII CCD diffractometer	4397 independent reflections
Radiation source: fine-focus sealed tube	3245 reflections with *I* > 2σ(*I*)
Graphite monochromator	*R*_int_ = 0.055
φ and ω scans	θ_max_ = 30.1°, θ_min_ = 2.0°
Absorption correction: multi-scan (*SADABS*; Bruker, 2009)	*h* = −18→18
*T*_min_ = 0.853, *T*_max_ = 0.968	*k* = −6→6
13545 measured reflections	*l* = −35→35

### Refinement

**Table:**

Refinement on *F*^2^	Primary atom site location: structure-invariant direct methods
Least-squares matrix: full	Secondary atom site location: difference Fourier map
*R*[*F*^2^ > 2σ(*F*^2^)] = 0.068	Hydrogen site location: inferred from neighbouring sites
*wR*(*F*^2^) = 0.168	H atoms treated by a mixture of independent and constrained refinement
*S* = 1.08	*w* = 1/[σ^2^(*F*_o_^2^) + (0.0606*P*)^2^ + 3.359*P*] where *P* = (*F*_o_^2^ + 2*F*_c_^2^)/3
4397 reflections	(Δ/σ)_max_ = 0.001
203 parameters	Δρ_max_ = 0.53 e Å^−^^3^
0 restraints	Δρ_min_ = −0.56 e Å^−^^3^

### Special details

**Table:**

Experimental. The crystal was placed in the cold stream of an Oxford Cryosystems Cobra open-flow nitrogen cryostat (Cosier & Glazer, 1986) operating at 100.0 (1) K.
Geometry. All esds (except the esd in the dihedral angle between two l.s. planes) are estimated using the full covariance matrix. The cell esds are taken into account individually in the estimation of esds in distances, angles and torsion angles; correlations between esds in cell parameters are only used when they are defined by crystal symmetry. An approximate (isotropic) treatment of cell esds is used for estimating esds involving l.s. planes.
Refinement. Refinement of F^2^ against ALL reflections. The weighted R-factor wR and goodness of fit S are based on F^2^, conventional R-factors R are based on F, with F set to zero for negative F^2^. The threshold expression of F^2^ > 2sigma(F^2^) is used only for calculating R-factors(gt) etc. and is not relevant to the choice of reflections for refinement. R-factors based on F^2^ are statistically about twice as large as those based on F, and R- factors based on ALL data will be even larger.

### Fractional atomic coordinates and isotropic or equivalent isotropic displacement parameters (Å^2^)

**Table:**

	*x*	*y*	*z*	*U*_iso_*/*U*_eq_	
Cl1	0.46301 (6)	0.58083 (17)	1.15354 (3)	0.02081 (18)	
Cl2	0.13465 (6)	−0.0073 (2)	1.03531 (3)	0.0258 (2)	
O1	0.36215 (16)	−0.0699 (5)	1.02088 (9)	0.0167 (4)	
N1	0.32626 (19)	0.3664 (5)	1.04970 (10)	0.0128 (5)	
C1	0.3584 (2)	0.3527 (7)	1.15041 (12)	0.0160 (6)	
C2	0.3352 (2)	0.2594 (8)	1.19925 (13)	0.0221 (7)	
H2A	0.3758	0.3204	1.2339	0.026*	
C3	0.2519 (3)	0.0759 (8)	1.19680 (14)	0.0247 (7)	
H3A	0.2357	0.0096	1.2299	0.030*	
C4	0.1922 (3)	−0.0109 (7)	1.14604 (14)	0.0234 (7)	
H4A	0.1355	−0.1375	1.1444	0.028*	
C5	0.2158 (2)	0.0887 (7)	1.09772 (13)	0.0186 (6)	
C6	0.3005 (2)	0.2685 (6)	1.09881 (11)	0.0136 (5)	
C7	0.3562 (2)	0.1873 (6)	1.01372 (11)	0.0123 (5)	
C8	0.3845 (2)	0.3240 (6)	0.96339 (11)	0.0125 (5)	
H8A	0.3733	0.5311	0.9645	0.015*	
H8B	0.4592	0.2910	0.9649	0.015*	
C9	0.3199 (2)	0.2055 (6)	0.90964 (11)	0.0128 (5)	
C10	0.3612 (2)	0.0027 (7)	0.88140 (12)	0.0156 (6)	
H10A	0.4293	−0.0667	0.8965	0.019*	
C11	0.3044 (2)	−0.1058 (7)	0.83020 (12)	0.0183 (6)	
H11A	0.3343	−0.2474	0.8114	0.022*	
C12	0.2067 (2)	−0.0057 (7)	0.80799 (12)	0.0177 (6)	
H12A	0.1691	−0.0769	0.7735	0.021*	
C13	0.1607 (2)	0.2029 (6)	0.83576 (12)	0.0151 (6)	
C14	0.0590 (2)	0.3104 (7)	0.81287 (12)	0.0189 (6)	
H14A	0.0213	0.2416	0.7782	0.023*	
C15	0.0150 (2)	0.5103 (8)	0.83999 (13)	0.0213 (6)	
H15A	−0.0527	0.5800	0.8240	0.026*	
C16	0.0700 (2)	0.6148 (7)	0.89197 (13)	0.0199 (6)	
H16A	0.0387	0.7521	0.9110	0.024*	
C17	0.1686 (2)	0.5172 (7)	0.91479 (12)	0.0171 (6)	
H17A	0.2052	0.5902	0.9494	0.021*	
C18	0.2165 (2)	0.3100 (6)	0.88767 (12)	0.0140 (5)	
H1N1	0.332 (3)	0.543 (8)	1.0462 (14)	0.014 (9)*	

### Atomic displacement parameters (Å^2^)

**Table:**

	*U*^11^	*U*^22^	*U*^33^	*U*^12^	*U*^13^	*U*^23^
Cl1	0.0224 (3)	0.0174 (4)	0.0219 (4)	−0.0038 (3)	0.0033 (3)	−0.0046 (3)
Cl2	0.0198 (3)	0.0294 (5)	0.0285 (4)	−0.0066 (3)	0.0058 (3)	−0.0084 (3)
O1	0.0234 (10)	0.0077 (10)	0.0203 (10)	0.0022 (8)	0.0077 (8)	−0.0007 (8)
N1	0.0208 (11)	0.0034 (12)	0.0152 (11)	0.0017 (9)	0.0057 (9)	0.0020 (9)
C1	0.0189 (13)	0.0116 (14)	0.0176 (13)	0.0024 (11)	0.0045 (10)	0.0000 (11)
C2	0.0277 (15)	0.0230 (18)	0.0161 (14)	0.0069 (14)	0.0062 (11)	0.0013 (13)
C3	0.0322 (16)	0.0236 (18)	0.0224 (15)	0.0066 (15)	0.0148 (13)	0.0064 (14)
C4	0.0271 (15)	0.0159 (16)	0.0317 (17)	0.0004 (13)	0.0163 (13)	0.0055 (14)
C5	0.0205 (13)	0.0154 (15)	0.0211 (14)	0.0029 (12)	0.0070 (11)	−0.0011 (12)
C6	0.0196 (13)	0.0059 (13)	0.0170 (13)	0.0030 (11)	0.0077 (10)	0.0021 (10)
C7	0.0132 (11)	0.0091 (13)	0.0142 (12)	0.0003 (10)	0.0021 (9)	−0.0017 (11)
C8	0.0161 (12)	0.0068 (12)	0.0148 (12)	−0.0002 (10)	0.0041 (10)	−0.0002 (10)
C9	0.0161 (12)	0.0091 (13)	0.0139 (12)	−0.0023 (11)	0.0051 (10)	0.0015 (11)
C10	0.0182 (12)	0.0120 (14)	0.0172 (13)	−0.0008 (12)	0.0052 (10)	0.0002 (11)
C11	0.0257 (14)	0.0131 (15)	0.0174 (13)	0.0008 (12)	0.0075 (11)	−0.0030 (12)
C12	0.0238 (14)	0.0140 (14)	0.0146 (13)	−0.0025 (12)	0.0032 (10)	−0.0021 (12)
C13	0.0192 (13)	0.0108 (14)	0.0154 (13)	−0.0045 (11)	0.0042 (10)	0.0009 (11)
C14	0.0200 (13)	0.0174 (16)	0.0175 (14)	−0.0017 (12)	0.0004 (11)	0.0010 (12)
C15	0.0151 (12)	0.0230 (17)	0.0248 (15)	0.0009 (12)	0.0029 (11)	0.0031 (13)
C16	0.0220 (14)	0.0157 (16)	0.0231 (15)	0.0017 (12)	0.0075 (11)	−0.0010 (12)
C17	0.0208 (13)	0.0136 (14)	0.0175 (13)	0.0006 (12)	0.0054 (10)	−0.0002 (12)
C18	0.0164 (12)	0.0098 (13)	0.0164 (13)	−0.0009 (11)	0.0053 (10)	0.0006 (11)

### Geometric parameters (Å, º)

**Table:**

Cl1—C1	1.738 (3)	C9—C10	1.371 (4)
Cl2—C5	1.734 (3)	C9—C18	1.436 (4)
O1—C7	1.227 (4)	C10—C11	1.418 (4)
N1—C7	1.354 (4)	C10—H10A	0.9500
N1—C6	1.418 (4)	C11—C12	1.367 (4)
N1—H1N1	0.85 (4)	C11—H11A	0.9500
C1—C2	1.390 (4)	C12—C13	1.416 (4)
C1—C6	1.394 (4)	C12—H12A	0.9500
C2—C3	1.390 (5)	C13—C18	1.426 (4)
C2—H2A	0.9500	C13—C14	1.426 (4)
C3—C4	1.390 (5)	C14—C15	1.364 (5)
C3—H3A	0.9500	C14—H14A	0.9500
C4—C5	1.390 (4)	C15—C16	1.420 (4)
C4—H4A	0.9500	C15—H15A	0.9500
C5—C6	1.398 (4)	C16—C17	1.375 (4)
C7—C8	1.528 (4)	C16—H16A	0.9500
C8—C9	1.520 (4)	C17—C18	1.416 (4)
C8—H8A	0.9900	C17—H17A	0.9500
C8—H8B	0.9900		
			
C7—N1—C6	122.0 (3)	C10—C9—C18	119.9 (3)
C7—N1—H1N1	120 (2)	C10—C9—C8	119.9 (3)
C6—N1—H1N1	117 (2)	C18—C9—C8	120.2 (2)
C2—C1—C6	122.1 (3)	C9—C10—C11	121.5 (3)
C2—C1—Cl1	119.1 (2)	C9—C10—H10A	119.3
C6—C1—Cl1	118.8 (2)	C11—C10—H10A	119.3
C3—C2—C1	119.2 (3)	C12—C11—C10	119.7 (3)
C3—C2—H2A	120.4	C12—C11—H11A	120.2
C1—C2—H2A	120.4	C10—C11—H11A	120.2
C4—C3—C2	120.2 (3)	C11—C12—C13	120.8 (3)
C4—C3—H3A	119.9	C11—C12—H12A	119.6
C2—C3—H3A	119.9	C13—C12—H12A	119.6
C3—C4—C5	119.6 (3)	C12—C13—C18	119.9 (3)
C3—C4—H4A	120.2	C12—C13—C14	121.4 (3)
C5—C4—H4A	120.2	C18—C13—C14	118.7 (3)
C4—C5—C6	121.6 (3)	C15—C14—C13	121.1 (3)
C4—C5—Cl2	118.1 (3)	C15—C14—H14A	119.5
C6—C5—Cl2	120.3 (2)	C13—C14—H14A	119.5
C1—C6—C5	117.3 (3)	C14—C15—C16	120.3 (3)
C1—C6—N1	120.8 (3)	C14—C15—H15A	119.9
C5—C6—N1	121.9 (3)	C16—C15—H15A	119.9
O1—C7—N1	122.7 (3)	C17—C16—C15	120.0 (3)
O1—C7—C8	121.2 (3)	C17—C16—H16A	120.0
N1—C7—C8	116.1 (3)	C15—C16—H16A	120.0
C9—C8—C7	111.9 (2)	C16—C17—C18	121.1 (3)
C9—C8—H8A	109.2	C16—C17—H17A	119.4
C7—C8—H8A	109.2	C18—C17—H17A	119.4
C9—C8—H8B	109.2	C17—C18—C13	118.8 (3)
C7—C8—H8B	109.2	C17—C18—C9	122.9 (3)
H8A—C8—H8B	107.9	C13—C18—C9	118.2 (3)
			
C6—C1—C2—C3	0.1 (5)	C18—C9—C10—C11	0.7 (4)
Cl1—C1—C2—C3	−179.7 (3)	C8—C9—C10—C11	−177.9 (3)
C1—C2—C3—C4	−0.5 (5)	C9—C10—C11—C12	0.5 (5)
C2—C3—C4—C5	−0.5 (5)	C10—C11—C12—C13	−0.8 (5)
C3—C4—C5—C6	1.9 (5)	C11—C12—C13—C18	−0.2 (5)
C3—C4—C5—Cl2	−176.1 (3)	C11—C12—C13—C14	179.6 (3)
C2—C1—C6—C5	1.2 (4)	C12—C13—C14—C15	179.7 (3)
Cl1—C1—C6—C5	−178.9 (2)	C18—C13—C14—C15	−0.5 (5)
C2—C1—C6—N1	−179.8 (3)	C13—C14—C15—C16	−0.2 (5)
Cl1—C1—C6—N1	0.1 (4)	C14—C15—C16—C17	0.9 (5)
C4—C5—C6—C1	−2.3 (4)	C15—C16—C17—C18	−0.8 (5)
Cl2—C5—C6—C1	175.8 (2)	C16—C17—C18—C13	0.1 (5)
C4—C5—C6—N1	178.7 (3)	C16—C17—C18—C9	179.0 (3)
Cl2—C5—C6—N1	−3.2 (4)	C12—C13—C18—C17	−179.6 (3)
C7—N1—C6—C1	117.3 (3)	C14—C13—C18—C17	0.5 (4)
C7—N1—C6—C5	−63.7 (4)	C12—C13—C18—C9	1.4 (4)
C6—N1—C7—O1	0.6 (4)	C14—C13—C18—C9	−178.4 (3)
C6—N1—C7—C8	−178.3 (2)	C10—C9—C18—C17	179.4 (3)
O1—C7—C8—C9	57.7 (3)	C8—C9—C18—C17	−2.0 (4)
N1—C7—C8—C9	−123.3 (3)	C10—C9—C18—C13	−1.7 (4)
C7—C8—C9—C10	−99.9 (3)	C8—C9—C18—C13	176.9 (3)
C7—C8—C9—C18	81.5 (3)		

### Hydrogen-bond geometry (Å, º)

**Table:**

*D*—H···*A*	*D*—H	H···*A*	*D*···*A*	*D*—H···*A*
N1—H1*N*1···O1^i^	0.84 (4)	2.00 (4)	2.823 (3)	165 (3)
C8—H8*A*···O1^i^	0.99	2.37	3.242 (4)	146
C8—H8*B*···O1^ii^	0.99	2.53	3.488 (4)	163

Symmetry codes: (i) *x*, *y*+1, *z*; (ii) −*x*+1, −*y*, −*z*+2.
